# 

**Published:** 1882-04

**Authors:** 


					﻿VOL/II.
APRIL, 1882.
NO. 4.
JRHE
Homeopathic physician
A MONTHLY JOURNAL OF MEDICAL SCIENCE.
“ Magna est veritas, el prcevalebit.”
Hl. O’. jLjHIHj, LIVE. ZD.
EDITOR.
CONTENTS:
(See inside Of Cover.)
PHILADELPHIA:
No. 3109 CHESTNUT STREET.
x. o nsr id o 3sr.
ALFRED HEATH & CO., Sole Agents for Great Britain,
114 Ebury Street, Eaton Square.
Yearly Subscription, $2.00, invariably in advance.-
. Entered at the Philadelphia Post-Office, as Second-Class Mall Matter* *'
				

